# Pancreatic Neuroendocrine Tumors—Diagnostic Pitfalls of Non-Diabetic Severe Hypoglycemia: Literature Review and Case Report

**DOI:** 10.3390/diagnostics15030337

**Published:** 2025-01-31

**Authors:** Simona Georgiana Popa, Andreea Loredana Golli, Cristina Florentina Matei, Alexandra Nicoleta Sonei, Cristin Vere, Radu Cimpeanu, Marian Munteanu, Alexandru Munteanu

**Affiliations:** 1 Department of Diabetes, Nutrition and Metabolic Diseases, University of Medicine and Pharmacy, 200349 Craiova, Romania; simona.popa@umfcv.ro; 2 Department of Public Health and Healthcare Management, University of Medicine and Pharmacy, 200349 Craiova, Romania; 3 Department of Diabetes, Nutrition and Metabolic Diseases, Emergency County Hospital, 200642 Craiova, Romania; cristinaflorentinamatei94@gmail.com (C.F.M.); alexandra.sonei@yahoo.com (A.N.S.); 4 Department of Gastroenterology, University of Medicine and Pharmacy, 200349 Craiova, Romania; cristin.vere@umfcv.ro (C.V.); radu.cimpeanu@umfcv.ro (R.C.); 5 Department of Surgery, University of Medicine and Pharmacy, 200349 Craiova, Romania; marian.munteanu@umfcv.ro (M.M.); alexandru.munteanu@umfcv.ro (A.M.)

**Keywords:** insulinoma, pancreatic neuroendocrine tumor, non-diabetic hypoglycemia, neuroglycopenic symptoms, diagnostic pitfalls

## Abstract

**Background**: Hypoglycemia in the case of persons without diabetes is a rare event, being usually, initially misinterpreted based on the symptoms that can mimic various diseases, especially of a neuro-psychiatric nature. In the case of the identification of insulin-mediated hypoglycemia, the evaluation of pancreatic neuroendocrine tumors, which represent the most common and worrisome causes of non-diabetic insulin-mediated hypoglycemia, must be considered. **Case Report**: We present the case of a 57-year-old patient, hospitalized for a history of approximately one month of recurrent episodes of symptoms suggestive for severe hypoglycemia. The biological evaluation performed during an episode of hypoglycemia showed a plasma glucose value of 44 mg/dL, insulinemia 16.3 µU/mL, C peptide 3.72 ng/mL, HbA1c 4.99%, absence of urinary ketone bodies and anti-insulin antibodies <0.03 U/mL. The CT and MRI examination showed a 15.3/15 mm rounded tumor in the pancreatic corporeo-caudal region. The pancreatic tumor formation was enucleated and the histopathological and immunohistochemical analysis confirmed the diagnosis of the pancreatic neuroendocrine tumor with a positive reaction for chromogranin A, synaptophysin and insulin, without malignancy features (Ki 67 positive in 1% of the tumor cells). The postoperative evolution was favorable, without episodes of hypoglycemia, the fasting insulinemia one day after surgery being 4.1 µU/mL and HbA1c at three weeks postoperatively being 5.51%. **Conclusions**: The management of patients with hyperinsulinemic hypoglycemia secondary to insulinoma involves multidisciplinary collaboration with an important role in recognizing symptoms suggestive of hypoglycemia in a person without diabetes, initiating biological and imaging evaluation, establishing the optimal therapeutic option and histopathological confirmation.

## 1. Introduction

Neuroendocrine tumors (NETs), first described in the pulmonary tissue, are rare and heterogeneous types of tumors; they could be localized in all organs that present diffuse neuroendocrine system, originating from the endodermal cells, depending on the primitive gut, and being classified in foregut (thymus, esophagus, lung, stomach, duodenum and pancreas), midgut (appendix, ileum, cecum and ascending colon) and hindgut (distal bowel and rectum) [1,2,3]. The gastrointestinal tract is the most frequently affected site (>60%), followed by the lung (>10%) and other sites less frequently affected such as the head, thyroid, breast and genitourinary system [3]. Pancreatic NETs (P-NETs) are relatively rare types of NET, being classified in insulinoma (I-oma), gastrinoma, Vasoactive Intestinal Peptide tumors (VIPoma), pancreatic polypeptide secretion tumors (PPoma), glucagonoma and somatostatinoma [3,4].

Severe hypoglycemia is frequently encountered in patients with diabetes treated with insulin or insulin secretagogues, but in patients without diabetes, hypoglycemia is unusual, and the symptomatology is often misinterpreted [5,6]. In this context, the urgent initiation of the clinical, biological and imaging evaluation of the patient is required in order to establish the etiological diagnosis and the optimal therapy to avoid the serious consequences of severe hypoglycemia [7,8].

The first step of the etiological diagnosis of hypoglycemia in patients without diabetes involves establishing whether the hypoglycemia is mediated by endogenous hyperinsulinism or other mechanisms. In the case of the identification of insulin-mediated hypoglycemia, the evaluation of pancreatic neuroendocrine tumors (P-NETs), which represent the most common and worrisome causes of non-diabetic insulin-mediated hypoglycemia, must be considered.

P-NET represents 30% of gastroenteropancreatic neuroendocrine tumors, most of them being non-functional and only 10–30% being functional, secreting one or more hormones or amines that determine suggestive clinical symptomatology [9].

In order to confirm the etiology of endogenous hyperinsulinemic hypoglycemia, the appropriate pathological diagnosis of P-NET should include the functional evaluation by specific staining for peptide hormones, especially insulin, but also serotonin and other amines depending on the clinical symptoms [9,10,11].

The identification of P-NET as the cause of severe hypoglycemia requires urgent therapeutic intervention for the remission of hypoglycemic episodes and their consequences and to increase the patient's quality of life.

## 2. Materials and Methods

In the current review, we emphasize the pitfalls in the identification and etiological diagnosis of hypoglycemia in non-diabetic individuals. Thus, we present the case of a female patient with small size P-NET, who presented with severe hypoglycemia. Our case study also highlights the role of the multidisciplinary team, and especially of the general practitioner, in recognizing the symptoms consistent with hypoglycemia, evaluating the patient and establishing the positive diagnosis of hypoglycemia and the etiological diagnosis, in order to optimally manage non-diabetic hypoglycemia and also to avoid diagnostic pitfalls.

### Aim

All procedures performed in studies involving human participants were in accordance with the ethical standards of the institutional review board (Report no. 35510/13 August 2024).

To carry out this narrative review, the PubMed database was analyzed, using the keywords “neuroendocrine tumors”, “insulinoma” and “hyperinsulinemic non-diabetic hypoglycemia”, and selecting publications such as case reports, clinical practice guidelines, original articles and reviews in English, which were available as full text.

## 3. Results

The 57-year-old patient was admitted to the Diabetes Clinic from Craiova Emergency Clinical Hospital in November 2021 for recurrent episodes of dizziness, fatigue, cold sweat, tremors, changes in behavior and anxiety, sometimes associated with seizures or deterioration of consciousness. The patient did not experience weight gain, palpitations, headaches, diarrhea, flushing or itching. Clinical symptoms occurred daily, for about 1 month.

The patient also had a history of hospital admissions in the Psychiatric Clinic due to repeated episodes of psychiatric symptoms such as behavioral disorders, psychomotor agitation and anxiety. During the last hospitalization in the Psychiatry Clinic, in the context of loss of consciousness, a capillary blood glucose level of 19 mg/dL was detected.

The patient denied alcohol consumption or intentional or accidental administration of medications that could induce hypoglycemia (insulin, sulfonylureas). The patient has no hereditary or personal history of diabetes, non-islet cell tumors, multiple endocrine neoplasia syndromes, gastric bypass surgery or any other significant diseases and was not on any prescribed medication.

A physical exam indicated overweight (body mass index: 25.55 kg/m^2^) and abdominal obesity (waist 90 cm), without detecting other significant clinical changes.

Starting from admission to Diabetes Clinic, the patient presented recurrent episodes of hypoglycemia (plasma glucose value being less than 55 mg/dL), unrelated to food intake, accompanied by confusion, dizziness, cold sweats, tremor, unresponsive to the ingestion of carbohydrates, which required the initiation of intravenous boluses of 33% glucose solutions (30–50 mL during episodes of severe hypoglycemia) and continuous intravenous infusion with 10% glucose solutions (on average 350 g glucose/24 h from glucose solutions administered i.v.). Symptoms of hypoglycemia remitted, and glycemic values normalized during continuous intravenous infusion of 10% glucose solutions, but the trend of glycemia rapidly decreased after the interruption of the i.v. administration of Glucose, despite the daily intake of 300 g of carbohydrates divided into 6–7 meals. However, two plasma glycemic values of ≥200 mg/dL (fasting plasma glucose 209 mg/dL and 3h postprandial plasma glucose 227 mg/dL) were randomly detected in the absence of the i.v. administration of glucose solutions.

The paraclinical evaluation during an episode of hypoglycemia indicated a plasma glucose value of 44 mg/dL, serum insulin level 16.3 µU/mL (normal 2.2–24.9 µU/mL), C peptide level 3.72 ng/mL (normal 1.1–4.4 ng/mL), HbA1c 4.99% (normal 4–5.6%), the absence of urinary ketone bodies and anti-insulin antibodies <0.03 U/mL (normal <0.4 U/mL). HbA1c was evaluated 7 months before admission, during routine investigations, and was 5.1%.

Based on the paraclinical explorations, other potential causes of hypoglycemia were excluded such as adrenal insufficiency (ACTH and serum cortisol levels in a sample taken at 8.00 a.m. were normal), thyroid dysfunction (normal TSH, FT4, FT3), organ failure (normal kidney and liver function) and sepsis (normal blood counts, normal inflammatory tests), respectively. The ophthalmoscopy, abdominal ultrasound, cranial and chest computed tomography (CT) examination were normal.

The clinical and biochemical data presented above suggested endogenous insulin-mediated hypoglycemia; therefore, P-NET was suspected and abdominal imaging investigations were performed.

**Figure 1:**
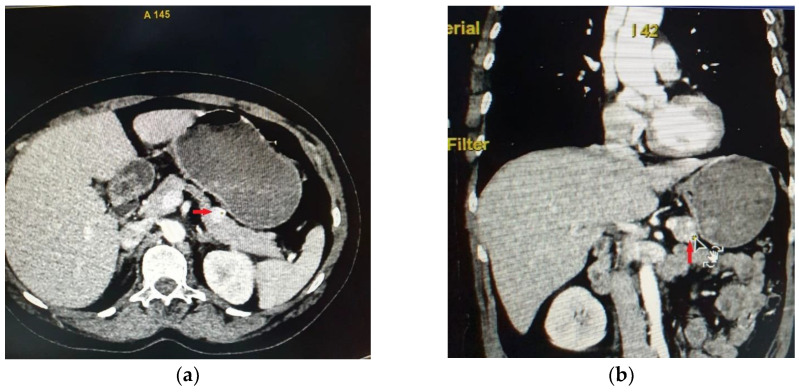
Abdominal CT-axial section (**a**) and coronal section (**b**), indicating that in the anterior part of the pancreatic corporeo-caudal region, there is an isodense tumor measuring 15.3/15 mm with intense iodophilia.

Abdominal CT showed in the anterior part of the pancreatic corporeo-caudal region an isodense rounded tumor measuring 15.3/15 mm, with intense iodophilia that is maintained in the venous phase (Figure 1). A complementary abdominal magnetic resonance imaging (MRI) was performed, indicating a pancreatic hypervascular tumor that appeared with hypo/isosignal at T1-weighted sequences and slightly hypersignal at T2-weighted sequences, homogeneous in the post-contrast study, with diffusion restrictions in diffusion-weighted imaging sequences correlated with apparent diffusion coefficient maps.

**Figure 2:**
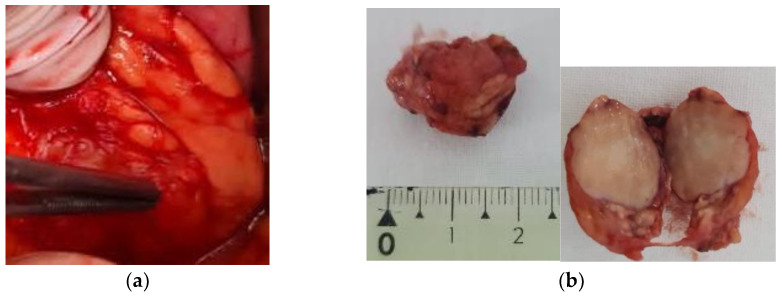
Intraoperative appearance of the tumor (**a**) and resected specimen with homogenous pattern (**b**).

The patient underwent tumor enucleation from the pancreatic corporeo-caudal region. A 1.5 cm, well-defined, yellow-gray tumor with elastic consistency, was excised (Figure 2). Visual and palpatory examination of the pancreas revealed no other pancreatic lesions.

Intraoperatively, the capillary glucose trend was monitored at 15-min intervals, with the adjustment of the intravenous 10% glucose solution infusion rate, based on the evolution of the glycemic profile. Shortly after the excision of the tumor, the glycemic values increased (226 mg/dL) in the absence of intravenous administration of glucose solutions. The postoperative evolution was favorable, without the occurrence of episodes of hypoglycemia. The fasting insulinemia determined on the second postoperative day was 4.1 µU/mL.

The histopathological examination highlighted a fragment of pancreatic tissue showing tumor proliferation consisting of round, large, relatively monomorphic cells, with hyperchromic excentric nuclei and abundant granular cytoplasm with a solid and trabecular pattern, rare mitoses (<2 mitoses/10 HPF) and desmoplastic stroma.

Regarding the immunohistochemical examination, specific staining was performed to confirm the diagnosis of P-NET. Thus, to establish the neuroendocrine origin, staining was performed for chromogranin A and synaptophysin, both reactions being positive. Also, the functional character of the tumor was analyzed, with the tumor cells being positive for insulin and negative for glucagon. The Ki67 index was analyzed as a tumor proliferation marker, with the index being positive in 1% of tumor cells. Immunohistochemical tests supported the diagnosis of P-NET G1, according to the WHO 2019 classification for gastroenteropancreatic neuroendocrine neoplasms [11].

Seven days after the surgical intervention, the patient was discharged asymptomatic and with normal glycemic profiles. HbA1c at three weeks postoperatively was 5.51%. Nine months postoperative, the patient is still free of all the previous hypoglycemic symptoms, with normal glycemic values and glycated hemoglobin being increased to 6.2% and the insulin level was 7.8 μU/mL.

The clinical, biological, imaging, histological and immunohistochemical findings support the diagnosis of functional well-differentiated P-NET insulinoma, confined to the pancreas, grade G1 according to the WHO criteria [11,12,13,14,15].

## 4. Discussion

Hypoglycemia recognition and prompt intervention are a unitary medical challenge, due to the life-threatening risks of hypoglycemia. Hypoglycemia should be suspected if the elements of the Whipple triad are identified in a patient without diabetes: venous blood glucose level <55 mg/dL (<3 mmol/L) (determined at the time of spontaneous development of symptoms, if feasible), the presence of specific signs and symptoms for hypoglycemia and their remission after carbohydrate intake [16].

In order to establish the etiological diagnosis of hypoglycemia, the anamnesis and clinical examination must take into account the information regarding the main potential causes of hypoglycemia, such as the lifestyle pattern (inanition, type, amount and food sources of ingested carbohydrates, abuse of alcohol or drugs); surreptitious, malicious, accidental or iatrogenic administration of hypoglycemic drugs (insulin, sulfonylureas, etc); and personal and hereditary medical history including diseases such as diabetes, cancer, psychiatric diseases, bariatric surgery, critical illness (hepatic, renal or cardiac failure), sepsis, hormone deficiency (glucagon, cortisol, growth hormone), non-beta cell tumors, Familial Endocrine Tumor Syndrome (FETS)-like multiple endocrine neoplasia type 1 or 4, neurofibromatosis type I or tuberous sclerosis complex [17,18,19]. Important to mention is “paraneoplastic hypoglycemia” which is related to other tumors like hepatocellular carcinomas, hemangiopericytomas, mesotheliomas, fibrosarcoma and gastrointestinal stromal tumors that secret insulin-like hormones, leading to hypoglycemia [20].

Regardless of the triggering cause of hypoglycemia, the signs and symptoms characteristic of hypoglycemia are the same and are divided into two categories: autonomic (palpitations, tremors, anxiety, pallor, tachycardia, hypertension, cold sweat, imperious hunger, paresthesia) and neuroglycopenic (headache, dizziness, visual impairment, behavioral disturbances, confusion, drowsiness, convulsions, coma) [5,6,21]. Since the initial symptoms would be varied and non-specific, mimicking various cardiovascular, endocrinological, neurological and psychiatric conditions, a multidisciplinary approach to making a detailed differential diagnosis is absolutely necessary for the correct management of hypoglycemia [21].

The diagnosis of hypoglycemia is performed by determining the plasma levels of glucose, markers of endogenous insulin synthesis and secretion (proinsulin, insulin, C-peptide) and beta-hydroxybutyrate, during a hypoglycemic episode occurring spontaneously or induced by a mixed meal or during a fast of up to 72 h. The biological evaluation should include a hormonal profile to identify potential hormonal deficiencies that could justify hypoglycemia and insulin antibodies.

Insulin-mediated hypoglycemia is defined based on glycemia ≤ 55 mg/dL, insulinemia ≥ 3 µU/mL, C-peptide levels ≥ 0.6 ng/mL, proinsulinemia ≥ 5 pmol/L and β-hydroxybutyrate levels ≤ 2.7 mmol/l [13]. Previously, the insulinemia threshold value for the diagnosis of hyperinsulinemic hypoglycemia was 5 µU/mL, but subsequent studies have indicated that in a significant percentage of patients; insulinoma remains undetected if a higher insulinemia cut-off value is used [22].

The peculiarity of the presented case is the association of neuroglycopenic symptoms of hypoglycemia not preceded by autonomic hypoglycemic symptoms, with values of insulinemia and C peptide higher than the threshold for defining insulin-mediated hypoglycemia, but not so high compared to the severity and persistence of hypoglycemia and refractoriness of hypoglycemic episodes to carbohydrate ingestion and parenteral glucose administration. 

Unlike most cases of insulinoma in which the presence of hypoglycemia is associated with significantly elevated levels of C-peptide and insulinemia, in our case, although the hypoglycemia was severe, frequent and relatively refractory to intravenous glucose administration, the C-peptide level did not increase in parallel with the severity of symptoms and hypoglycemia [23,24].

The appearance of neuroglycopenic symptoms not preceded by autonomic ones is most likely explained by the presence of hypoglycemia-associated autonomic failure due to the recurrent severe hypoglycemia episodes [25].

The association of severe hypoglycemia with levels of insulinemia and C peptide higher than the diagnostic threshold value of insulin-mediated hypoglycemia but not significantly increased can be justified by the challenging coexistence of insulinoma and type 2 diabetes which was masked by insulinoma [23,26]. The association of type 2 diabetes in our patient is suggested by the random plasma glucose values higher than 200 mg/dL and also by the occurrence of persistent hyperglycemia after the surgical removal of the insulinoma. Persistent hyperglycemia following surgical removal of the insulinoma may suggest underlying diabetes (the patient had glycemic values > 200 mg/dL prior to surgery) but it may also be the consequence of damage to the pancreas during the surgical procedure, which caused secondary hyperglycemia (HbA1c nine months postoperatively was 6.2%) [26]. 

Considering that the level of C peptide above 6.1 ng/mL has increased specificity and sensitivity (96% and 100%, respectively), together with the size of the tumor above 2.6 cm in the prediction of malignancy, the value of C peptide in the presented patient can be explained by the benign nature of the insulinoma, confirmed by the pathological examination [27].

In spite of much about the pathogenesis of P-NETs being largely unknown, approximately 10% are part of the FETSs [28,29,30] and the treatment is targeted taking into account the cellular type and hormonal profile. The risk factors for developing a P-NET are body mass index, smoking, alcohol consumption, genetic factors (-1031 TC and CC genotype distribution of TNF-α promoter polymorphism and IL1β-511 for single-nucleotide polymorphism), family history of cancer (especially of P-NET) and first-degree family-history of esophageal cancer, adding to the risk factors other types of cancer, like gallbladder, gastric or ovarian cancer and sarcoma and personal history of chronic pancreatitis [31,32,33,34,35,36,37,38].

If P-NETs are suspected as a cause of hypoglycemia, the diagnosis and management should be based on key features of neuroendocrine neoplasm represented by proliferative activity, tumor growth rate using response evaluation criteria in solid tumors, locoregional and systemic extent of the disease and somatostatin receptor expression [9,11].

The diagnosis of P-NETs includes anamnestic data related to symptomatology suggestive of a certain type of functional P-NET and personal or family history of NETs or conditions frequently associated with NETs, non-invasive imaging evaluation such as CT or MRI and invasive imaging evaluation—endoscopic ultrasonography (EUS). Confirmation of the functional character of P-NET is performed through the immunohistochemical examination of the tumor tissue.

The appropriate pathological (histopathology and immunohistochemistry) diagnosis of P-NET should include the functional evaluation by specific staining for peptide hormones (insulin, serotonin and other amines depending on the clinical symptoms), Chromogranin A and synaptophysin and the establishment of tumor grade by reporting the ki67 index and the mitotic count per 2 mm^2^ [9,10,11].

I-oma, the most current form of P-NETs, first described in 1927 in a murine model, is the most common and the most frequently functioning endocrine pancreatic tumor [39]. I-omas are very rare functioning P-NETs, with a reported incidence of 1–32 new cases/106 population/year [14]. Generally, I-oma’s are benign, solitary, intrapancreatic lesions, with 0.5–2 cm dimension at the moment of diagnosis [17,40], but there are sometimes some exceptions. Although the I-oma are typically localized in the pancreatic tissue, ectopic positions have been described, such as: duodenal wall, spleen, perisplenic tissue, duodenohepatic ligament and the surrounding tissue of the pancreas [41,42]. Also, rare associations with I-oma have been presented such as pregnancy, type 2 diabetes, the transformation of metastatic non-functioning P-NET into I-oma and until now, eleven giant I-oma (majority with pancreatic tail localization) [43,44,45,46,47,48]. Also, for functional NET, which is a hormone-secreting tumor, it is necessary to dosage the non-specific (Chromogranin A, Neuron-Specific Enolase, pancreatic polypeptide, Human Chorionic Gonadotropin and α-fetoprotein) and specific (Serotonin, Gastrin, Insulin, Glucagon, Somatostatin and Vasoactive Intestinal Peptide) neuroendocrine markers, for I-oma being recommended Chromogranin A, Neuron-Specific Enolase and Insulin [49,50,51].

Among the imaging methods for insulinoma detection, CT represents the first line of evaluation with a sensitivity varying between 30 and 80% depending on the size of the tumor, followed by MRI with a sensitivity of 85–92% for MRI [52,53]. Insulinoma detection sensitivity increases significantly to 90% when findings from both MRI and CT are combined [53].

While MRI is more useful in detecting pancreatic tumors smaller than 2 cm in size, CTs are useful for identifying tumor metastasis, but also for detecting a potential association of neuroendocrine neoplasms with non-neuroendocrine neoplasms such as acinar cell carcinoma, adeno-carcinoma, squamous cell carcinoma and an association known as mixed neuroendocrine/non-neuroendocrine neoplasm (MiNEN) [11,15,52,53,54].

However, EUS had higher sensitivity (40–92.6% depending on the location of the tumor) than CT and MRI because of its ability to detect small I-omas, even infracentimetric tumors [55,56,57,58].

In general, the imaging identification of insulinoma is difficult due to its small size, thus more complex diagnostic methods such as echo-endoscopy or scintigraphy with indium-labeled GLP-1 receptor agonists are frequently required [24]. For our patients, the imaging diagnosis of insulinoma was not relatively difficult, the tumor being observed in abdominal CT, with MRI being performed complementary only to confirm the presence of the tumor.

The immunohistochemistry feature is vital for diagnosis, through Chromogranin A, Synaptophysin, insulin and the ki67 index [59,60]. Islet-1 combined with CDX2 expression is positive in approximately 90% of cases [61].

Referring to the therapeutic possibilities, there are supportive measures (dietary—glucose-rich meals or nocturnal tube feeding for superior control of hypoglycemia episodes), medical treatment (diazoxide for blocking the ATP-dependent kalium channels of the pancreatic β cells, combined with a thiazide diuretic for fluid retention prevention), somatostatin receptor ligands (SRLs) and sometimes glucocorticoids [4,62,63,64,65,66,67,68,69,70,71,72]. A lot of drugs are used for metastatic or inoperable aggressive forms of I-oma, like SRLs peptide receptor radionuclide therapy, m-TOR (Everolimus) and tyrosine kinase (Sunitinib) inhibitors and also cytotoxic chemotherapy [4,62,67,73,74,75,76,77,78,79]. A conservative follow-up therapy is recommended for non-functioning P-NETs [4,80].

For reduced dimensions of I-oma, endoscopic or Surgery (I.E., Pancreaticoduodenectomy, debulking) methods are recommended, with specific peculiarities—depending on grading and functioning or not functioning type [4,67,81,82,83]. Surgery resection or methods of embolization or radioablation are recommended in liver metastases from I-oma (which could be up to 78% of cases). [4,62,67,79,84].

Complications of I-oma are described after surgery (general complications which can appear after an intervention and subphrenic abscess, pancreatic fistula, acute pancreatitis) and in correlation with neuroendocrine tumor disease, like carcinoid crisis and tumor necrosis [85,86]. Although I-oma presents a favorable prognosis, surveillance is classicly correlated with tumor dimension (≥2 cm), metastases, ki-67 proliferation index and age [59,87].

Hypoglycemia is a potentially life-threatening pathology; therefore, it is very important to recognize the suggestive symptoms in time and to establish the etiological diagnosis of hypoglycemia in order to be able to intervene effectively therapeutically.

Regardless of the cause, severe, recurrent, untreated hypoglycemia can have cardiovascular consequences such as arrhythmias, QT interval prolongation, myocardial infarction or stroke, but also neurological damage, progressive personality changes, memory disorders, psychosis, progressive dementia or sudden death [7,13].

Diagnosing insulinoma is challenging due to the rarity of this type of P-NET and the nonspecific clinical manifestations that can mimic various conditions and can be misinterpreted, thus establishing the diagnosis depends on a detailed evaluation of the clinical data, the heredo-collateral and personal pathological history and a high index of suspicion. For these reasons, the time between the onset of symptoms and the establishment of the diagnosis is generally long, sometimes years, and patients may refer to several different health care providers before receiving the correct diagnosis. 

Hence, future studies and guidelines should attempt to delineate and compile the clinical, biological, histological and genetic features that will prompt the clinician to suspect P-NETs.

The current case report highlights these diagnostic difficulties and draws attention to the importance of interdisciplinary collaboration and complete and consistent evaluation of patients who present with symptoms suggestive of hypoglycemia, apparently unexplained by an obvious pathology.

P-NET serves as a topic of a spectrum of further extensive research that will allow a better understanding of histological features and, principally the identification of a novel drug target, permitting the development of precision therapy tailored to the patient's individual characteristics.

## 5. Conclusions

The presented case highlights the pitfalls in the interpretation of the signs and symptoms of hypoglycemia, especially in the case of people without diabetes, and the need to confirm it by determining the glycemic value at the respective moment, followed by an etiological diagnosis as early as possible in order to correct the underlying cause.

Thus, the management of patients with hyperinsulinemic hypoglycemia secondary to insulinoma involves multidisciplinary collaboration with an important role in recognizing symptoms suggestive of hypoglycemia in a person without diabetes, initiating biological and imaging evaluation, in order to classify, stage the disease and confirm the etiology of endogenous hyperinsulinemic hypoglycemia, establishing the optimal therapeutic option and histopathological confirmation.

The identification of P-NET as the cause of severe hypoglycemia requires urgent therapeutic intervention for the remission of hypoglycemic episodes and their consequences and to increase the patients' quality of life.

## Data Availability

No new data were created or analyzed in this study. Data sharing is not applicable to this article.
